# Excess Body Mass—A Factor Leading to the Deterioration of COVID-19 and Its Complications—A Narrative Review

**DOI:** 10.3390/v13122427

**Published:** 2021-12-03

**Authors:** Weronika Gryczyńska, Nikita Litvinov, Bezawit Bitew, Zuzanna Bartosz, Weronika Kośmider, Paweł Bogdański, Damian Skrypnik

**Affiliations:** 1 Faculty of Medicine, Poznan University of Medical Sciences, 61-701 Poznan, Poland; sspwera@wp.pl (W.G.); nikitogco@gmail.com (N.L.); bezawitdemeke@gmail.com (B.B.); nowaczyk.zuzanna@gmail.com (Z.B.); weronika.kosmider@gmail.com (W.K.); 2 Ethiopian Medical Students’ Association, Zambia Street, Addis Ababa P.O. Box 9302, Ethiopia; 3 Department of Treatment of Obesity, Metabolic Disorders and Clinical Dietetics, Poznan University of Medical Sciences, 60-569 Poznan, Poland; pbogdanski@ump.edu.pl

**Keywords:** SARS-CoV-2, metabolic syndrome, severity of COVID-19, physiopathology, obesity, excess body mass

## Abstract

Currently, the world is facing two serious pandemics: obesity and COVID-19. It is well-established that the prevalence of obesity has risen dramatically, causing a deterioration in the health quality of the population and increasing susceptibility for the unfavourable course of acute infections. It has been observed that excess body mass significantly influences the COVID-19 outcome. The aim of this review is to present the latest scientific reports on the impact of excess body mass on the course and complications of COVID-19. The Web of Science, PubMed, and Google Scholar databases were searched. Only studies reporting patients stated to be COVID-19 positive based on the results of a nasopharyngeal swab and the ribonucleic acid test were included. It is shown that thromboembolic and ischemic complications, namely stroke, disseminated intravascular coagulation, severe hyperglycaemia, and leukoencephalopathy are more likely to appear in COVID-19 positive patients with obesity compared to non-obese subjects. COVID-19 complications such as cardiomyopathy, dysrhythmias, endothelial dysfunction, acute kidney injury, dyslipidaemia, lung lesions and acute respiratory distress syndrome have a worse outcome among obese patients.

## 1. Introduction

Currently, worldwide medicine is managing two serious pandemics: obesity and COVID-19. Based on the body mass index (BMI), in 2016, the World Health Organisation (WHO) estimated that 1.9 billion adults were overweight, of which 650 million were obese; together, this accounted for 39% and 13% of the global population, respectively [1]. The WHO reports that 84.78 million COVID-19 infections were confirmed, and 1.85 million associated deaths recorded by 6 January 2021 [2].

Obese or overweight patients infected with COVID-19 have an increased risk of intubation and death compared to those with a normal BMI [3,4,5,6,7]. Research reports that people with obesity are more contagious and for longer than people with normal body weight in conditions of SARS-CoV-2 infection. According to Ablashir, obesity prolongs the excretion of the virus from the body by 42% and delays the ability to produce interferons [5].

Obesity, as one of the greatest factors of complications due to COVID-19, has been proved to worsen the overall outcome of patients. Data analysis showed that individuals with obesity are at higher risk for COVID-19, resulting in a 48% higher morbidity and mortality rate compared to those whose BMI is described as within the normal range [8]. Therefore, the costs of treatment are also remarkable. According to studies during the first wave of COVID-19 in Europe, the direct financial responsibility associated with this disease was estimated at 13.9 billion EUR, of which 76% was spent on people with overweight and obesity [9]. The course of COVID-19 and its complications in patients with obesity are serious clinical problems which need current knowledge to succeed. Highlighting the importance of some of the crucial recommendations and explanations, this paper helps to better understand the course of COVID-19 and the treatment choices in excess body mass patients.

The aim of this paper is to present the latest reports on the impact of excess body mass on the course and complications of COVID-19. We raise aspects related to the nervous system, respiratory system, circulatory system, excretory system, coexisting diabetes of overweight and obese people during the course of COVID-19. Thus, the paper will show the unique cross-talk of the two greatest world pandemics.

## 2. Materials and Methods

### 2.1. Eligibility Criteria

Studies included in this review were published from 1 December 2019 to 30 September 2021. They are investigations on the causative associations between COVID-19 and obesity, by reported measures of body mass index (BMI) over 30 kg/m^2^ defined as obesity by WHO and obesity classified as over BMI ≥ 28 kg/m^2^ for Asians according to the Regional Office for the Western Pacific (WPRO).

Original articles considering humans, other animals, in vitro tests and published in English were included. Review papers and case reports were only included when the crucial information could not be found in any other available resources. During the research, articles with data duplication, case reports, non-English or published before December 2019 were removed. 

All of the patients in the included studies were COVID-19 positive based on positive results from a nasopharyngeal swab using the ribonucleic acid test.

### 2.2. Selection Process

The databases searched a total of 1206 records. After reading the abstracts, 1066 studies were removed. The browsing led to the elimination of 57 papers, leaving a total of 83. This was done according to the eligibility criteria. The screening was mechanically done by checking the date of publication, BMI of subjects included in the studies, and if patients included in the studies tested positive for COVID-19 from a nasopharyngeal swab using the ribonucleic acid test.

### 2.3. Data Collection Process

This review was conducted in accordance with Preferred Reporting Items for Systematic Reviews and Meta-Analyses (PRISMA). Researchers utilized articles published in the Web of Science (“all databases” search), PubMed, and Google Scholar databases. The following keywords were used without other restrictions in study design or language: “COVID-19” or “2019-nCoV” or “coronavirus” or “coronavirus disease 2019” or “corona-virus disease” or “novel coronavirus” or “2019-nCov”or “novel coronavirus infection” or “2019-nCov infection” or “severe acute respiratory syndrome coronavirus 2” or “SARS-CoV-2”) AND (“obesity” or “extra body mass” or “overweight” or “obese” or “BMI” or “body mass index” or “excessive body mass” or “visceral fat” or “excessive fat” or “abdominal fat” or “visceral adipose tissue” or “visceral adiposity” or “central adiposity” or “waist circumference”). The flow diagram of the review is presented in Supplementary Figure S1.

### 2.4. Outcome Measures

The outcome measure was the association between obesity and SARS-CoV-2 as manifested by increased mortality, hospitalization and severe complications.

## 3. Results

### 3.1. Cardiovascular Complications

#### 3.1.1. Cardiomyopathy

When identifying individuals who are susceptible to worse complications from C0VID-19 infection, the demographics include obese individuals [10]. Cardiomyopathy in obese patients stems from an increase in total blood volume and cardiac output because of the high metabolic activity of excessive fat [11]. Due to this, left ventricular hypertrophy or dilation and increased left ventricular wall stress are observed. There is an increased predisposition to cardiomyopathy in COVID-19 infected patients with obesity [12]. Myocardial injury in COVID-19 patients is marked due to high-sensitivity troponin I above the threshold of 28 pg/mL [13].

Another state leading to cardiomyopathy in COVID-19 patients is a complication from the treatments utilised. Chloroquine is used to inhibit SARS-CoV-2 by increasing the endosomal pH, in turn creating an unfavourable environment for viral replication. The drug also blocks virus receptor binding by altering the terminal glycosylation of angiotensin-converting enzyme 2 (ACE2). Cardiotoxicity manifestations of this drug are restrictive or dilated cardiomyopathy or conduction abnormalities [14].

Other risk factors for cardiomyopathy in obese COVID-19 patients include an increased level of stress because of the social and economic issues arising from the pandemic—Takutsobo or stress cardiomyopathy. As this relates to the COVID-19 pandemic and stress cardiomyopathy, there was a case report on two elderly Hispanic patients. The first patient suffered from anxiety leading her to presenting of heart failure symptoms; the second patient developed stress cardiomyopathy due to increased emotional stress as an indirect consequence of the ongoing pandemic [15].

#### 3.1.2. Dysrhythmias

Hypoxia and inflammatory stress can lead to dysrhythmias in COVID-19 patients [13]. A study showed that dysrhythmias were present in 17% of hospitalised and 44% of intensive care unit (ICU) patients with COVID-19 [16]. The most frequently observed dysrhythmia is sinus tachycardia, which is attributed to hypovolemia, hypoperfusion, hypoxia, elevated body temperature, pain, and anxiety. 

Obese patients with dysrhythmias associated with an elevation in serum troponin should be strictly monitored as there might be an onset of myocardial injury, acute myocarditis and acute coronary syndrome (ACS). In addition to the direct result of COVID-19 infection, treatments for this viral illness can lead to dysrhythmias. Azithromycin interferes with protein synthesis and binds to the 50s ribosome. The drug might lead to development of dysrhythmias, especially in obese patients [13].

#### 3.1.3. Thromboembolic Complications

Among the various cardiovascular complications, there is a growing occurrence of thromboembolic complications in hospitalised COVID-19 patients. This event has been contributing to increased morbidity and mortality. In autopsies, an increased level of D-dimer has been observed, which is a reflection of the inflammatory and pro-coagulant state. In these patients, a clear reduction of fibrinolysis caused coagulopathy. A hypercoagulable state due to hypofibrinolysis was found [17]. To better understand the statistics behind this complication, an analysis was performed on data gathered from in-hospital patients. Out of 388 patients, thromboembolic events occurred in 28 [18]. Out of the 388 patients, 130 patients had a BMI higher than or equal to 25 kg/m^2^, 144 patients had a BMI from 25–30 kg/m^2^ and 87 patients had a BMI higher than or equal to 30 kg/m^2^. There was no association found between the BMI of these patients and the likeliness of this complication occurring. However, a pre-disposed state, obesity, clearly shows a favouring of thromboembolic complications [19].

#### 3.1.4. Disseminated Intravascular Coagulation

In obese individuals, there is a predisposition to a pro-inflammatory state, even before SARS-CoV-2 infection. This is because of the heightened level of inflammatory cytokines secreted by excess fat. The inflammatory cytokines can prompt tissue factor production on endothelium and monocytes causing coagulation, as blood coming into contact with tissue factor brings this on [20].

When analysing a study of 361 subjects, in which 39.8% had a BMI of 25–30 kg/m^2^ and 24.1% a BMI of higher than or equal to 30 kg/m^2^, disseminated intravascular coagulation (DIC) was seen in 8 patients (2.1%). Seven (88%) patients with over-disseminated intravascular coagulation (DIC) died during hospitalisation [18]. It can be assumed that the increased inflammatory state associated with obesity might have been a factor in the development of this complication. 

#### 3.1.5. Ischemic Complications

Ischemic complications, namely stroke, are associated with COVID-19 infection. The complication is often associated with abnormal coagulation, including elevated levels of D-dimer and fibrinogen. There have been studies analysing infection cases in which ischemic conditions have occurred [21]. These studies attribute hypercoagulability to ischemic conditions. However, the exact process remains unknown.

A particular study was done on patients from 312 hospitals in 46 states with regard to ischemic stroke. Analysis performed on patients presenting with ischemic pre-COVID stroke included 27,991 (16.8%) obese patients out of 166,586, while 517 of the 2086 COVID-19 infected patients were obese. The research led to the conclusion that ischemic complications are more likely to be seen in male, younger and obese patients. Multiple conditions, such as diabetes, acute renal failure, acute coronary syndrome, venous thromboembolism, intubation, and comorbid intracerebral or subarachnoid haemorrhage, were also cited in regard to this complication [22].

#### 3.1.6. Endothelial Dysfunction

As seen in previous viral infections, a pathway leading to endothelial dysfunction, SARS-CoV is no different. Obesity is closely related to this complication. Endothelial dysfunction is mainly associated with the entry point of SARS-CoV-2, which is the angiotensin-converting enzyme 2 (ACE2) receptor, located on the surface of endothelium and other cells. The range of features, in regard to endothelial dysfunction, was observed on post-mortem histological analysis in COVID-19 infection. The first was severe endothelial injury associated with intracellular SARS-CoV-2 virus. The second feature was related to vascular thrombosis with microangiopathy and the occlusion of alveolar capillaries. The third was the rise of new vessels by the mechanism of intussusceptive angiogenesis in the lungs [23].The above-stated features outline a clear correlation between COVID-19 and endothelial dysfunction and its consequences. Moreover, it can be hypothesised that pre-existing endothelial dysfunction in obese patients exacerbate the already deteriorating condition. Summary of cardiovascular complications of COVID-19 in patients with obesity is presented in Table 1.

### 3.2. Respiratory Complications 

Obesity may constitute a risk of respiratory failureduring the course of infection caused by SARS-CoV-2 virus, and potentially exacerbaterespiratory complications arelated to SARS-CoV-2 infection should be considered in several aspects. Regardless of the region of the world and the size of the study groups, a relationship was observed between a high BMI and the frequency of intubation of patients with COVID-19 undergoing treatment in intensive care units [5,7,24,25,26,27]. It has also been shown that the results of the chest computed tomography (CT) examination in obese COVID-19 patients were characterised by much worse results compared to those with normal body weight [28,29,30,31]. An example is a French cohort study involving 124 patients with COVID-19, where 75.8% of intensive care unit (ICU) patients had a BMI >30 kg/m^2^, and patients showing a BMI > 35 kg/m^2^ needed orotracheal intubation and mechanical ventilation 7.36 times more often than those showing a BMI *<* 25 kg/m^2^ [32]. A study of 95 Wuhan COVID-19 patients classified according to BMI displayed a higher percentage of changes in CT examination in people with obesity, manifested by areas of ground-glass opacity associated with partial filling of the alveoli with fluid (ascending 100% for people with obesity in relation to 94.9% for patients with normal body weight), a crazy-paving pattern resulting from swelling of the alveolar walls (30.6% to 23.7%), enlarged or increased number of pulmonary mediastinal lymph nodes (33.3% to 10.2%) and pleural effusion (11.1% to 10.2%), which presents heavier lung lesions [28].

“Cytokine storm” means an overactive immune response, manifested by the release of cytokines in amounts that are harmful to the body. Acute phase cytokines that appear minutes to hours after infection include tumour necrosis factor (TNF) and interleukin 1 beta (IL-1β) as well as interleukin 8 (IL-8) and monocyte chemoattractant protein-1 (MCP-1), which are chemotactic cytokines. Subsequently, the level of interleukin (IL-6) increases, the production of which is stimulated by TNF and IL-1β, and then interleukin 10 (IL-10) appears, indicating that the body is trying to control the inflammatory response [33].

Acute respiratory distress syndrome (ARDS), related by a “cytokine storm” damaging the respiratory epithelium, is a significant cause of deaths associated with SARS-CoV-2 infection [7,32,34,35]. Obesity promotes hyperactivation of the complement system, which may be a factor causing sequelae inflammatory processes resulting in a cytokine storm [24,25]. Obesity is accompanied by chronic inflammation associated with hypoxia and adipocyte dysfunction, which resultsinappearance of pro-inflammatory cytokines such as TNF-alpha, IL-6, MCP-1, IL-1 beta and IL-17A, likely to play a role in lung injury associated with ARDS [6,7,34].

In addition, people with obesity have a decreased concentration of adiponectin, which has anti-inflammatory properties, and an increased concentration of leptin, which has pro-inflammatory properties [36], as well as an increase in acute phase agents such as C-reactive protein (CRP) and amyloid antigen [4]. The above-mentioned pro-inflammatory factors promote the increased involvementof immune cells (including macrophages, and T and B lymphocytes) [5,28]. These obesity-accompanying factors result in an impaired immune response and may affect the lung parenchyma, predisposing to damage to the alveolar-capillary barrier and the translocation of protein-rich fluid into the respiratory system. Filling the alveoli with fluid reduces the compliance of the respiratory system and leads to hypoxemia. Pulmonary fibrosis occurs in the subsequent stages of ARDS [3,37]. Leija-Martínez et al. observed association between increased levels of TNF-alpha and IL-17 A in the blood serum of people with obesity and high predisposition to ARDS [7].

Obesity is also associated with overexpression of ACE2, a functional receptor used by the SARS-CoV-2 virus to invade cells [24,35]. Ablashir observed that the engagement of SARS-CoV-2 initialises spiking protein (proteins that allow the SARS-CoV-2 virus to enter host cells and induce infection) to the ACE2 receptor, which occurs with membrane-bound ACE2 down-regulation. Angiotensin II (AngII), the substrate for ACE2, can accumulate due to the down-regulation of ACE2 activity in the lungs. High levels of AngII can proceed to the increase in neutrophil growth, highervascular permeability and the aggravation of pulmonary oedema, which will finallyresult in ARDS [5,34,36]. Zhou et al. assumed that ACE2 overexpression in obese people is likely to be a factor in an increased vulnerability to COVID-19, as well as a tendency to an increased risk of acute respiratory failure [24]. Kruglikov and Scherer suspect that the interaction of adipocytes and pulmonary lipofibroblasts with the SARS-CoV-2 virus through the involvement of ACE2 receptors may lead to the progression of pulmonary fibrosis and thus worsen the course of COVID-19 [38].

Excess visceral adipose tissue significantly affects the mechanics and physiology of the respiratory system, which increases the tendency of people with obesity to respiratory failure when undergoing COVID-19. Fat tissue accumulating in the abdominal cavity, as well as around the ribs and diaphragm, significantly reduces the compliance of the chest wall, which significantly reduces the overall compliance of the respiratory system, as well as the forced expiratory volume in 1 s (FEV-1) and forced vital capacity (FVC), which is also due to increased pressure in the abdominal cavity [5,36]. In addition, obesity is a mechanical difficulty in imaging diagnostics, both due to the difficulties in positioning and transporting the patient by medical personnel, and the limitations of medical equipment [5]. An example is the problem of excessive body weight during pulmonary ultrasound, as the adipose tissue dampens the ultrasound waves, making the image less clear and more difficult to interpret [6].

Excess fat tissue in the upper respiratory tract can lead to airway obstruction, which promotes hypoxia [4]. Anatomical changes in the head and neck area resulting from excess fat tissue also make intubation difficult [5]. The inequality of ventilation and perfusion accompanying obesity results in reduced oxygen saturation in the blood; in addition, oxygen consumption for the work of the respiratory system in people with obesity is disproportionately high, which leads to obese patients having lower oxygen reserves. Such conditions require fast intubation, despite the mechanical difficulties [5].

Patients with obesity suffer from obstructive sleep apnoea, which is associated with impaired lung function through impaired pulmonary carbon monoxidetransmission and increased inflammation, which may also increase the risk of life-threatening respiratory failure in the course of COVID-19 [6,34,39,40].People with obesity also suffer from respiratory muscle failure (reduced diaphragm contractility) [4], as well as increased airway resistance, which translates into increased respiratory work and an increased energy expenditure needed to perform it. In addition, oxygen consumption for the work of the respiratory system in people with obesity is relatively high [5,24].

The clinical data to date indicate a greater predisposition of people with obesity to the development of ARDS in the course of COVID-19 and disease progression [26,31,40]. A study conducted in Italy, involving 242 patients with COVID-19, showed that among patients with ARDS admitted to the ICU, the median BMI was 27.7 kg/m^2^, which suggests that overweight is associated with an increased risk of disease progression. Despite the theory of the “obesity paradox” appearing in publications, i.e., the protective effect of obesity on mortality in the course of ARDS [26], there are no clear data indicating a higher or lower mortality rate in people with obesity in relation to patients with normal body weight burdened with ARDS in the course of COVID-19 admitted to the ICU. However, it should be remembered that the aspects of obesity’s influence on respiratory complications in COVID-19 discussed in this chapter may affect the rapid course of the disease as well as diagnostic difficulties, which could potentially affect the initiation of therapy at the optimal time and its success. Summary of respiratory complications of COVID-19 in patients with obesity is presented in Table 2.

### 3.3. Renal Complications

Xu et al. found the expression of ACE2 within the kidneys to be comparable to that of the lungs. Besides, renal cells were found to be especially rich in ACE2 and transmembrane protease, serine 2 (TMPRSS; encodes a protein that belongs to the serine protease family) genes, the main targets for SARS-CoV-2. Alongside the role of ACE2 in helping viral entry to the kidney tissues plays a significant role in COVID-19-induced kidney injury [41].

Xiao et al. inspected how hypertension, its major metabolic co-phenotypes, and antihypertensive medicines relate to ACE2 renal expression utilizing data from up to 436 patients whose kidney transcriptomes were characterised by RNA-sequencing. The results indicated that hypertension or antihypertensive treatment can hardly modify the expression of the key entry receptor for SARS-CoV-2 within the human kidney. Further information considers that in the absence of SARS-CoV-2 infection, kidney ACE2 is more likely nephro-protective, but the age-related increment of its expression in lungs and kidneys may be significant to the chance of SARS-CoV-2 infection [42].

Moreover, acute kidney injury is frequent in SARS-CoV-2-associated disease, and perscient to multi-organ dysfunction in SARS-CoV-2 infection. SARS-CoV-2 might also actuate kidney injury and provoke histopathological abnormalities, including podocytopathy, microangiopathy and acute tubular necrosis [43].

A prospective cohort study of 1603 patients with a confirmed COVID-19 infection reported that the main related conditions on admission were hypertension (35.7%), obesity (20.3%), and diabetes (15.2%). In result, 11.4% of the patients developed AKI during their hospital admission. Studies have shown that patients with developed AKI have a higher mortality rate than those without renal complications [44].

Gabrielle Page-Wilson et al.’s retrospective cohort study of 1019 SARS-CoV-2 positive adults admitted to an academic medical center, where the prevalence of overweight and obesity was 75.2%, initialreports of hospitalised patients shown a dominance of obesity and high rates of acute kidney injury, hemodialysis, shock and intubation [45].

Another retrospective case series included 327 patients hospitalised with confirmed COVID-19, commonly observed comorbid conditions on the admission were obesity (34.6%), diabetes (42.5%), hypertension (63.9%) and hyperlipidaemia (34.9%). In 137 of 179 (76.5%) patients with observed AKI, AKI was present on admission and another group of 26 (14.5%) patients developed AKI after 48 h of admission. Patients with AKI were significantly older and had a higher prevalence of major comorbid conditions (obesity, diabetes, hyperlipidaemia, hypertension and chronic kidney disease (CKD)). This study showed that patients with AKI when compared with patients without AKI (58.1% (104 of 179) vs. 19.6% (29 of 148)) mortality rate was significantly higher [46].

A study of the Italian National Institute of Health (Istituto Superiore di Sanità, ISS) among patients with confirmed COVID-19 showed that obesity was present in 411 (11.1%) of the cases of death. It was found that patients who died due to COVID-19 were significantly younger than patients without obesity and had a higher predominance of acute renal failure and superinfection. Superinfection is a process by which a cell that has already been contaminated by one infection is co-infected with another virus later. Further analysis revealed the association of obesity with acute renal failure, age *<* 60 and male gender [41].

A study by Rhian M Touyz et al. strongly indicates that the presence of AKI in patients with confirmed COVID-19 is associated with increased risk of mortality. Additionally, it seems likely that many patients with kidney disease will not return to their pre-COVID-19 renal function. Existing data suggest that 25–35% patients have not returned to baseline kidney function at the time of hospital discharge [47].

Based on these studies, obesity, diabetes, hypertension and hyperlipidaemia can be associated with developing AKI during hospital admission.

Moreover, in a population-based cohort study in England by Holman analysing COVID-19-related patients with type 1 and type 2 diabetes, more than half of the groups (62.3% and 55.4% accordingly) revealed renal impairment or cardiovascular disease. Renal impairment, male sex, older age, financial deprivation, non-white ethnicity, previous strokes and congestive heart failure are correlated with increased COVID-19-related mortality in both types of diabetes [48].

Furthermore, the renin–angiotensin–aldosterone system (RAAS), which is usually over-activated in patients with obesity, has been connected with SARS-CoV-2 infection. In view of this contemplation, RAAS should be considered as a mechanistic factor of specific significance in obesity. It drives COVID-19 severity via increased AngII signaling and the loss of ACE2/Mas. The MAS receptor is a G protein-coupled receptor, which connects to the angiotensin-II metabolite angiotensin (1–7). Clinical evidence about direct RAAS blockade in COVID-19 are not clear, the most recent work proposes an unbiased or even defencive effect that might emerge from AngII signaling inhibition [49].

In conclusion, results on relation RAAS targeting and COVID-19 infection are inconsistent, and this issue needs further investigation [49].So far, the results on relation RAAS targeting and COVID-19 infection are inconsistent, and this issue needs further investigation. Additionally, studies strongly indicate that obesity is connected with AKI development shortly after hospital admission and associated with increased risk of mortality. Based on this, AKI should be considered as a relevant risk factor for poor outcome of COVID-19. Summary of renal complications of COVID-19 in patients with obesity is presented in Table 3.

### 3.4. Metabolic Complications

#### 3.4.1. Diabetes

Patients hospitalised with COVID-19 presented hyperglycaemia. The meta-analysis with over 3700 patients shows a pooled proportion of 14.4% for newly diagnosed diabetes in hospitalised COVID-19 patients [50]. Furthermore, it was noticed that SARS-CoV-2 infection significantly worsens hyperglycaemia in patients with glucose metabolism disturbances [51]. SARS-CoV-2 manages infects and replicates in human beta-cells which express viral entry proteins. The virus generates a lower expression of insulin and a higher expression of glucagon. Induce pancreatic dysfunction leads to hyperglycemia or diabetes. Researchers also suggest cellular transdifferentiation [52,53,54]. Patients with obesity may suffer from this complication more often because obesity is associated with insulin resistance [55] and is a predictor of future diabetes [56]. The study by Santos et al. suggests that combining the cytokine storm noticed in COVID-19 and inflammation induced by insulin resistance provokes hyperglycemia in patients with obesity. [57]. Additionally, SARS-CoV-2 cell entry depends on ACE2. Therefore, the higher expression of ACE2 among obese patients could be the reason for pancreatic failure [58,59,60].

In a study comparing the clinical characteristics of patients with COVID-19, obese patients showed higher levels of blood glucose 5.61 mmol/L [(interquartile range) IQR, 4.2–14.51] vs. 4.86 [IQR, 3.95–13.49] than non-obese ones [28]. In another study, the baseline BMI was higher among hospitalised COVID-19 patients with newly diagnosed diabetes (94 patients with mean BMI 24.5 kg/m^2^) and hyperglycaemia (129 patients with mean BMI 24.4 kg/m^2^) [61]. The basic characteristics of 166 COVID-19 patients were divided into three groups: control, secondary hyperglycaemia and diabetes, showing a gradual increasing trend of BMI values among these groups [62].

The study of Zhu et al. analysed 293 patients with COVID-19 including mild and moderate cases (n = 217) and severe and critical cases (n = 76). The study shows that fasting blood glucose (FBG) was associated with BMI. The study excluded patients with diabetes, cancer, children, and included no one with end-stage chronic kidney disease, hepatic failure, hepatitis B, pancreatitis, haematological system diseases, cachexia, severe debilitating illness, and schizophrenia. The univariate logistic regression analysis demonstrated a significantly higher odds ratio (OR) of severe or critical condition in COVID-19 patients with elevated BMI (OR 1.570, 95%CI 1.199–2.056, *p* = 0.001). In comparison to mild and moderate cases, severe and critical patients had significantly higher FBG (5.30 mmol/L, IQR 4.80–5.90 vs. 7.35, IQR 5.60–9.58 mmol/L, *p* *<* 0.0001). [63]

Chen et al. performed a 6-month prospective study investigating clinical results of 64 hospitalized patients without diabetes. The results showed the risk of inducing insulin resistance among these patients. However, researchers could not eliminate BMI influencing carbohydrate metabolism, since the average BMI was higher than recommended. [64].

It is known that enterovirus infections, especially those due to group B coxsackieviruses can develop diabetes in humans [65,66]. Therefore, there is a possibility that COVID-19 may also induce diabetes. However, some studies indicate that newly diagnosed diabetes could have had its onset before COVID-19 infection, and the infection only unmasked the problem [67]. Patients with COVID-19 ≤ 60 years was more likely to present with abnormalities in glucose metabolism due to obesity [51]. Additionally, in COVID-19 patients, hyperglycaemia could be induced by steroid therapy [68] or caused by endogenous stress [69]. Moreover, glucose control could be inadequate due to the decreased quality of healthcare since health professionals are overwhelmed by the pandemic [70].

The risk of COVID-19 diabetes is unknown in non-hospitalised patients with obesity. There is no solid data that SARS-CoV-2 induces diabetes voluntarily. To solve these problems, the CoviDIAB project has been created to establish and characterize new-onset and COVID-related diabetes. (covidiab.e-dendrite.com) [71]. To fully explain the diabetes-induced by COVID-19 is necessary to perform long-term follow-up of children and adults [72].

#### 3.4.2. Dyslipidaemia

The “cytokine storm” elemental COVID-19 generates immune-mediated inflammatory dyslipoproteinemia, as a result of decreased specialized pro-resolving mediator biosynthesis causes elevated triglycerides, increased lipoprotein oxidation, also low HDL-C and LDL-C levels [73].Considering these results, it should be noted that, obese patients are prone to dyslipidaemia [74,75].Hypertrophic adipocytes create a state of systemic lipid imbalance [76]. The study of Aung et al. identified causal relationships between BMI, LDL cholesterol and susceptibility to SARS-CoV-2 infections [77]. In addition, another in silico and in vitro study suggests that ACE2 expression is increased in obese subjects which may be due to dysregulation in lipid metabolism increased ACE2 expression in obese subjects. The study of Zhu et al. investigated 489,769 individuals with COVID-19, of which 24% were obese (BMI ≥ 30.0 kg/m^2^). Obese patients had lower HDL-cholesterol compared to normal weight patients (49.5 vs. 62.6 mg/dL; *p* *<* 0.001), higher LDL (137.3 vs. 135.0 mg/dL; *p* *<* 0.001) and higher triglycerides (189.5 vs. 119.6 mg/dL; *p* *<* 0.001). There obese were at higher risk of developing severe COVID-19 [78]. Another study also showed that triglycerides (1.81 mmol/L [IQR 0.63–6.43] vs. 1.34 mmol/L [IQR 0.3–7.59]) and LDL (3.14 mmol/L [IQR 2.0–4.42] vs. 2.56 mmol/L [IQR 2.04–4.27]) were elevated to a much higher extent in obese patients compared to non-obesity patients [28]. In another trial a group of patients with obesity, compared to the normal-weight group, obese patients had higher serum triglycerides (70.4 vs. 105.4 mmol/L; *p* *<* 0.001), serum cholesterol (103.5 vs. 129 mmol/L; *p* *<* 0.001), LDL (57.6 vs. 87.11 mmol/L; *p* *<* 0.001) and lower HDL (49.4 vs. 41.9 mmol/L; *p* *<* 0.001) [79].

It is underlined that obese patients tend to have lower HDL-cholesterol levels [80] which was shown to be linked with a higher percentage of severe cases in COVID-19 [81]. COVID-19 patients in severe or critical condition, which BMI was higher than mild and moderate (23.43 kg/m^2^ vs. 25.11 kg/m^2^; *p* *<* 0.001) had significantly lower HDL levels (1.15 mmol/L [IQR 0.97–1.41] vs. 1.02 mmol/L [IQR 0.83–1.25], *p* *<* 0.0001)) compare to mild and moderate cases which indicate that HDL is protective factor for preventing COVID-19 exacerbation [63]. Another study showed that non-survivors have decreased HDL levels. The results demonstrated that dyslipidaemia is associated with the poor prognosis of COVID-19 and HDL levels have a consequential role [75].

However, the issue of the influence of COVID-19 on lipid profile needs further investigation, since obesity on its own causes severe impairment of systemic lipid homeostasis due to calorie excess. Additionally, during infection plasma triglyceride increases [82]. Summary of metabolic complications of COVID-19 in patients with obesity is presented in Table 4.

## 4. Discussion

In support of this research, we have been able to find evidence that links obesity with worse complications of COVID-19 infection. These complications can lead to serious conditions that threaten the lives of patients. In order to better understand the implications of obesity in COVID-19 patients, we divided the common complications seen in patients based on the targeted body systems.

Cardiovascular complications, such as cardiomyopathy, dysrhythmias, thromboembolic complications, disseminated intravascular coagulation, endothelial dysfunction and ischemic changes, have been associated with SARS-CoV-2 infection. Currently, the clear mechanism behind cardiac injury and COVID-19 infection is not known. However, we were able to find that there is growing evidence correlating the entry pathway of the virus to this specific complication. ACE2 is the host receptor for SARS-CoV-2, and the virus has a high affinity to it. It is also the receptor found in the heart, lungs, pericytes, and vessels. After the intertwining of the virus with the host receptor, the inflammatory state can lead to myocarditis, the loss of contractile function, altered ejection fraction, damage to cardiomyocytes and cause several cardiovascular problems. This process is magnified in obese patients because of ACE2 overexpression [5,34,37], an increased inflammatory state and excess adipose tissue. All these heightened changes in obese patients can cause severe cardiovascular complications such as endothelial dysfunction. Moreover, analysis of studies has shown obese patients are more susceptible to having a stroke during the prognosis of the infection [21].

In regard to respiratory complications, clinical data have shown that obese patients have required more care as seen by more frequent intubation [5,7,24,25,26,27] and worse imaging results like in a chest computed tomography [28,29,30,31]. We attribute these clinical results to different anatomical and physiological factors seen in obese individuals [4]. Excess fat tissue located in the upper respiratory tract has been seen to cause airway obstruction possibly leading to a hypoxic state. Additionally, excess fat found around respiratory muscles including diaphragm already put obese individuals at a disadvantage when it comes to normal ventilation. Physiologically, hyperactivation of the complement system is observed in obese patients which might lead to a cytokine storm which is already promoted by SARS-CoV-2 infection.

Renal complications such as RAAS targeting in COVID-19 infection are insufficient to indicate a strong influence on obese patients and need further investigation. However, studies strongly indicate that obesity, young age, high BMI and diabetes are connected with development AKI and superinfection shortly after hospital admission and associated with increased risk of mortality. Additionally, we found that patients with normal BMI have lower prevalence of developing renal complications than overweight patients. Furthermore, existing data suggest that 25–35% of patients who experienced renal complications will not gain their complete renal function when discharged from the hospital. Based on this evidence, existing or developed AKI has to be strongly connected with a high-risk factor of unsatisfactory outcome for COVID-19 patients with obesity.

Obesity has been linked to several metabolic disorders. From the gathered data, it can be seen that COVID-19 infection has been linked to diabetes which was newly diagnosed among hospitalised COVID-19 patients. Additionally, worsening of hyperglycemia in patients with already diagnosed glucose metabolism disturbances has been seen. We have concluded that obese individuals have a higher risk for developing metabolic disorders. Obese COVID-19 patients have an increased chance of developing these metabolic complications as compared to patients with a normal BMI. However, further research is recommended.

This particular research focuses on obese individuals and the severity of the complications of SARS-CoV-2 infection in these individuals. The entry pathway of the virus shows that any patient infected can develop these complications; however, hospitalisation and death rates are higher for patients with obesity [83]. It is also seen that these complications have worse progressions in obese patients. After separately examining several complications that can be seen in different body-systems and also the higher hospitalization and death rates, we were able to conclude that obesity is a condition that worsens the prognosis of COVID-19 causing higher risk for developing said complications and also a worse outcome because of the complications.

## 5. Limitations

The present study includes a large number of analyses; however, they differed in data quality. The groups of patients in studies differed from each other in terms of ethnicity, the severity of COVID-19, and comorbidities. Additionally, there is a lack of reliable data focused only on people with obesity. BMI is not a separate factor usually, patients with obesity suffer from other diseases like diabetes, hypertension, dyslipidemia, coronary heart disease which could strongly influence results. Furthermore, there is no data on mild and moderate cases of COVID-19, who were not included in the study. Their results could significantly influence the rate of post-COVID conditions. In addition, there are no long-lasting studies that analyzed COVID-19 complications in obese people. Further investigation should be conducted in this area.

## 6. Conclusions

It can be concluded that the ongoing pandemic of obesity has unfavourably contributed to the COVID-19 pandemic. Patients with obesity may experience complications from the virus on a more severe level due to multiple physiological changes that have been previously caused by the excessive amount of adipose tissue.

Among these complications, thromboembolic and ischemic complications, namely stroke, disseminated intravascular coagulation, worsened hyperglycaemia and leukoencephalopathy are more likely to appear in patients with obesity compared to the non-obese group. Furthermore, complications of COVID-19, such as cardiomyopathy, dysrhythmias, endothelial dysfunction, acute kidney injury, dyslipidaemia, lung lesions and ARDS, have a worse outcome among obese patients. Poor results in respiratory complications are mainly observed due to the obesity-related overexpression of ACE2, which is a functional receptor used by the SARS-CoV-2 virus to invade cells. This contributes to the increased susceptibility to COVID-19 and increases the risk of acute respiratory distress syndrome as well as pancreatic failure. There is still a vital need for further research into the relation between excess body mass and COVID-19 complications.

## Figures and Tables

**Table 1:** Summary of cardiovascular complications of COVID-19 in patients with obesity.

Complication	Methodology	Result	Citation
Cardiomyopathy	A comparative analysis done on obese and non-obese patients. The subject pool consisted of 357 hospitalised patients, from which 340 patients with confirmed, severe COVID-19 and 85 were obese.	The effect of obesity on the severity of COVID-19, including critical COVID-19.	[10]
	Data from Korean National Health Insurance Service, including 28,679,891 people that didn’t show prevalent hypertrophic cardiomyopathy.	28,679,891 people that didn’t show prev An association was confirmed between body mass index (BMI) and prevalence of clinical hypertrophic cardiomyopathy.	[11]
	In vitro, NHBE cells from non-obese (BMI *<* 30 kg/m^2^) and obese (BMI ≥ 30 kg/m^2^) were obtained.	cardiomyopathy The underlying mechanism for increased severity of COVID-19 complications in obese individuals might be attributed to dysregulated lipogenesis and high ACE2.	[12]
	A search using PubMed and Google Scholar. The authors used keywords like to refine the search “COVID-19”, “SARS-CoV-2”, “myocardial injury”, “myocarditis”, “acute myocardial infraction”, “dysrhythmia”, “arrhythmia”, “heart failure”, “venous thromboembolism”, “coagulable”.	COVID-19 infections did lead to cardiovascular complications. Even though obese subjects were included in the study, a clear distinction between the complications and obesity was not made.	[13]
	Two cases of Takotsubo cardiomyopathy were examined. In both cases, the subjects were postmenopausal women.	During the COVID-19 pandemic, an increased incidence of stress cardiomyopathy has been reported.	[14]
	Analyses of other researches and a complete summary of the latest studies on cardiovascular complications arising from SARS-CoV-2 infection.	COVID-19 medications may increase the risk of cardiac complications. Physicians should be aware of this fact.	[15]
Dysrhythmias	Retrospective case study of 138 patients hospitalized at Zhongnan Hospital of Wuhan University in Wuhan, China, from 1 January to 28 January 2020; final date of follow-up was 3 February 2020.	A significant number, 41%, of the subjects received ICU care during the hospitalization while 4.3% of the subjects ended in death.	[16]
Thromboembolic complications	Blood was drawn from 40 critically ill COVID-19 patients and ROTEM was performed.	A hypercoagulable state due to severe hypofibrinolysis.	[17]
	A study on 388 patients with laboratory-proven COVID-19 admitted to a university hospital in Milan, Italy. From 361 subjects, 39.8% had a BMI of 25–30 kg/m^2^ and 24.1% had a BMI of higher or equal to 30 kg/m^2^.	Outcomes were categorized as primary and secondary. The former included any thromboembolic complication, including venous thromboembolism, ischemic stroke, and acute coronary syndrome/myocardial infarction, while the latter included overt disseminated intravascular coagulation (DIC).	[18]
	A total of 9330 participants were divided into BMI quartiles at baseline.	Without the use of oral anticoagulants, it was shown that the 4th quartile as compared to other BMI quartiles had a significant increase of TEE.	[19]
Disseminated intravascular coagulation	A total of 88 patients with HFRS. Patients were stratified to groups with intravascular coagulation (n = 27) and patients that did not have intravascular coagulation. Extracellular vesicle tissue factor activity and other factors were measured.	Patients with intravascular coagulation had significantly higher peak extracellular vesicle tissue factor activity levels compared with those without intravascular coagulation.	[20]
Ischemic complication	The study reviewed multiple researches aiming to summarise the current status of research on COVID19, hypercoagulability and ischemic stroke.	Injury of endothelial cells was a direct result of the cytokine storm caused bySARS-CoV-2 invasion. Stroke seen in COVID-19 patient is a result of hypoxia caused by the infection.	[21]
	In this study, the subjects were 166,586 ischemic stroke controls and 2086 ischemic stroke-COVID from 312 hospitals in 46 states.	Increased morbidity and mortality is attributed to ischemic stroke in COVID-19 patients. This result was compared to patients from 2019 with ischemic stroke and pneumonia.	[22]

**Table 2:** Summary of respiratory complications of COVID-19 in patients with obesity.

Complication	Methodology	Result	Citation
Requirement for invasive mechanical ventilation	Retrospective cohort study analyzed results of 124 patients admitted in ICU patients for SARS-CoV-2.	75.7% ICU patients had a BMI > kg·m^−2^.	[32]
	A total of 242 patients with COVID-19 developed ARDS.	The median BMI among patients with ARDS admitted to the ICU was 27.7 kg/m^2^.	[26]
Changes in chest computer tomography	Clinical results of 95 patients with COVID-19.	Patients with obesity had a higher percentage of changes in CT examination.	[28]
Increased susceptibility to COVID-19	The study reviewed multiple researches argue that adipocytes and adipocyte-like cells play a role in pathogenic response to SARS-CoV-2.	Obesity and diabetes are potential comorbidities for COVID-19 infections, due to upregulated in adipocytes expression of ACE2.	[38]
	The study reviewed multiple researches illustrated the role of obesity in COVID-19	Obesity increases the susceptibility to COVID-19 and promotes the progression to respiratory failure.	[24]
Influence of obstructive sleep apnoea	An analysis of patients receiving mechanical ventilation with respiratory failure as a result of COVID-19.	Imparied lung function caused by sleep apnoea increases the risk of severe respiratory failure.	[39]

**Table 3:** Summary of renal complications of COVID-19 in patients with obesity.

Complication	Methodology	Result	Citation
Acute kidney injury	A total of 1603 Patients with a confirmed COVID-19 infection, the main related conditions on admission were obesity (20.3%), diabetes (15.2%) and hypertension (35.7%).	A total of 11.4% of the patients developed AKI during their hospital admission. The mortality rate in this group is higher than the worldwide.	[44]
	A total of 1019 SARS-CoV-2 positive adults with 75.2% prevalence of obesity and overweight.	Patients with obesity demonstrated higher rates of developing acute kidney injury, shock, intubation and hemodialysis.	[45]
	A total of 327 patients hospitalised with confirmed COVID-19, commonly observed comorbid conditions on the admission were obesity (34.6%), diabetes (42.5%), hypertension (63.9%) and hyperlipidaemia (34.9%).	Significantly higher mortality in patients with AKI.	[46]
	3694 of patients dying with COVID-19(411 presented obesity) in Italy were reviewed to extract information.	Obesity was associated with increased probability of experiencing acute renal failure and shock.	[41]
Renal impairment	A population-based cohort study of of COVID-19 related patients with diagnosed diabetes.	Increased mortality was associated with renal impairment of diabetes, higher BMI and glycaemic control.	[48]

**Table 4:** Summary of metabolic complications of COVID-19 in patients with obesity.

Complication	Methodology	Result	Citation
Diabetes	A total of 95 patients with COVID-19 were divided into the obesity group and non-obesity group based on their BMI.	In obesity group, blood glucose was higher than non-obesity one.	[28]
	A total of 453 patients with laboratory-confirmed COVID-19 infection were classified into four categories: normal glucose, hyperglycemia, newly diagnosed diabetes, diabetes.	Mean BMI of 129 patients with hyperglycemia was 24.4 kg/m^2^.	[61]
		Mean BMI of 94 patients with newly diagnosed diabetes was 24.5 kg/m^2^.	[61]
	A total of 166 COVID-19 patients were divided into three groups: control, secondary hyperglycemia with no diabetes history and patients with diabetes.	Gradual increasing trend of BMI values among these groups.	[62]
	The multivariate stepwise binary logistic analysis was used to test the dose-response effect of FBG levels on the risk of severe and critical conditions in COVID-19 patients.	Significantly higher fasting blood glucose (FBG) occured in severe and critical patients was associated with BMI.	[63]
	The prospective study investigated clinical results of 64 patients without diabetes diagnosed with COVID-19.	COVID-19 may increase the risk of insulin resistance in overweight patients without diabetes.	[64]
Dyslipidemia	Associations between obesity traits, quantitative cardiometabolic parameters and SARS-CoV-2 positivity in the UK Biobank cohort.	Casual relationships between BMI, LDL cholesterol and susceptibility to SARS-CoV-2 infection.	[77]
	Investigation of 489,769 patients with COVID-19.	Obese patients had lower HDL-cholesterol, higher LDL and higher triglycerides compared to normal weight patients. Obese patients were at higher risk of developing severe COVID-19.	[78]
	Analysis of clinical results of 95 patients with COVID-19 were divided into the obesity group and non-obesity group based on their BMI.	Elevated triglycerides and LDL cholesterol in obese patients compared to non-obesity patients.	[28]
	Analysis of clinical results of 230 COVID-19 patients were divided according to their body-mass index (BMI) into three groups: normal weight, overweight, and obese.	Higher serum triglycerides, serum cholesterol and lower HDL in a group of patients with obesity compared to the normal-weight group.	[79]
	Mean BMI of severe and critical condition COVID-19 patients was higher than mild and moderate states.	A significantly lower HDL levels in severe or critical patients.	[61]
	Retrospective analysis changes in lipid profiles and relationships with severity of disease in 216 COVID-19 patients.	Dyslipidemia is associated with the poor prognosis od COVID-19.	[75]

## Data Availability

The data that support the findings of this review are available from the corresponding author upon reasonable request.

## Supplementary Materials

The following are available online at https://www.mdpi.com/article/10.3390/v13122427/s1, Figure S1. Flow diagram of the review.

## Abbreviations

ACE2	Angiotensin-converting enzyme 2
ACS	Acute chest syndrome
AKI	Acute kidney injury
AngII	Angiotensin II
ARDS	Acute respiratory distress syndrome
BMI	Body mass index
CKD	Chronic kidney disease
CRP	C-reactive protein
CT	Computed tomography
DIC	Disseminated intravascular coagulation
DVT	Deep vein thrombosis
FBG	Fasting blood glucose
FEV-1	Forced expiratory volume in one second
FVC	Forced vital capacity
ICU	Intensive care unit
IL-1β	interleukin 1 beta
IL-6	interleukin 6
IL-8	interleukin 8
IL-10	interleukin 10
IL-17A	interleukin 17A
ISS	Istituto Superiore di Sanità
MCP-1	Monocyte chemoattractant protein-1
MR	Myocardial Infarction
RAAS	Renin–angiotensin–aldosterone system
TNF	Tumour necrosis factor
TMPRSS	Transmembrane protease, serine 2
WHO	World Health Organization
WPRO	Regional Office for the Western Pacific
