# Supplementary figures and images for: Excess Body Mass—A Factor Leading to the Deterioration of COVID-19 and Its Complications—A Narrative Review
Source: Viruses. 2021 Dec 3;13(12):2427. doi: 10.3390/v13122427 (PMC8708912; doi:10.3390/v13122427)

**Supplementary Figure S1.** Flow diagram of the review.

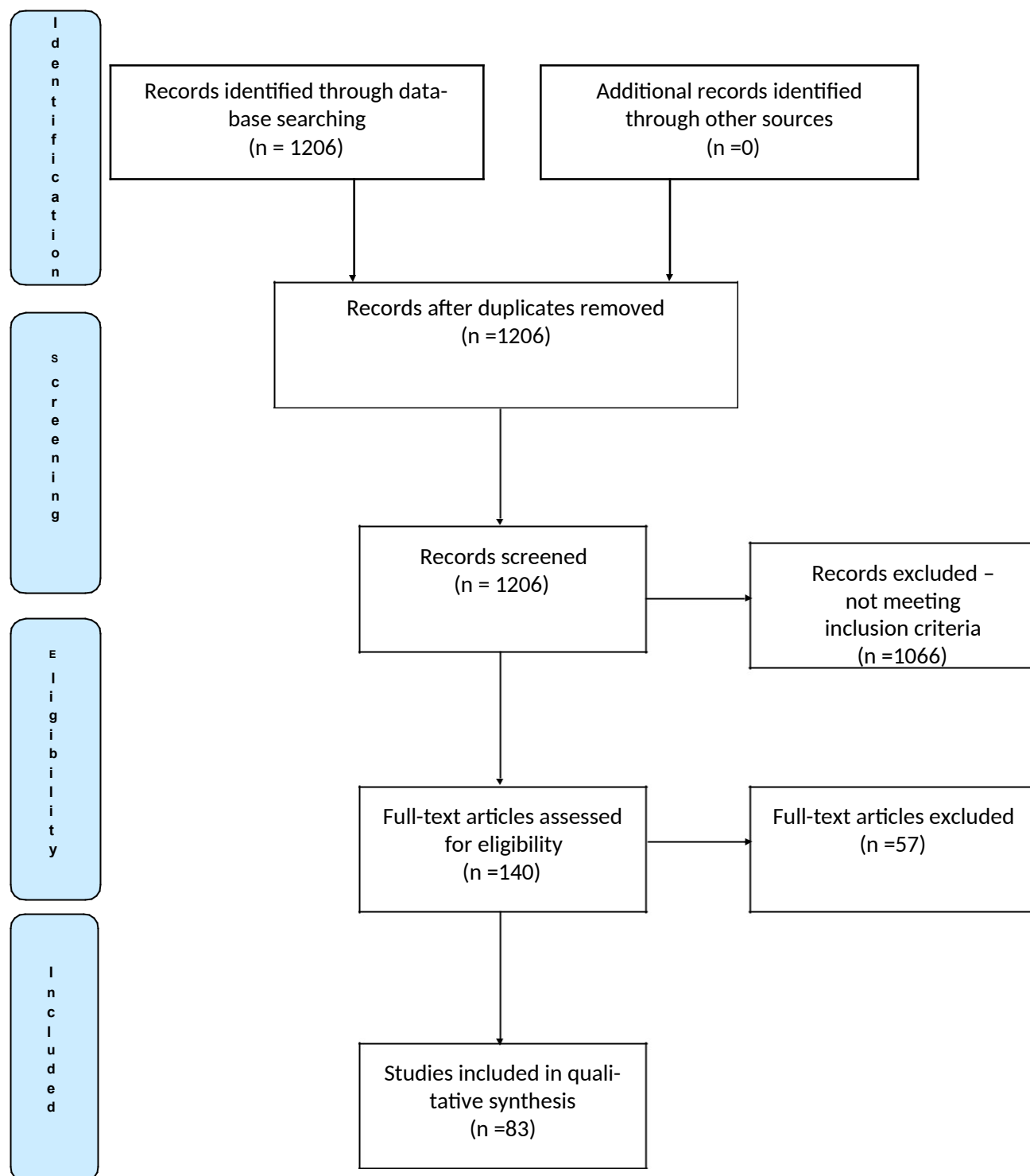

Supplement: Supplementary file 1 [file viruses-13-02427-s001.zip › viruses-1469631-supplementary.pdf]
